# Constructing a nomogram for ICSI-ET assisted reproductive outcomes in infertile men based on lifestyle and sperm quality: a multicenter retrospective survey

**DOI:** 10.3389/fendo.2025.1657141

**Published:** 2025-11-28

**Authors:** Ke Wang, Shihui Wang, Yan Xu, Jie Bai, Mengmeng Ma, Yuping Fan, Xin Huang, You Zhang, Ningxin Qin

**Affiliations:** 1 Center of Reproductive Medicine, Shanghai Key Laboratory of Maternal Fetal Medicine, Shanghai Institute of Maternal-Fetal Medicine and Gynecologic Oncology, Shanghai First Maternity and Infant Hospital, School of Medicine, Tongji University, Shanghai, China; 2 School of International Medical Technology, Sanda University, Shanghai, China; 3 Department of Nursing Administration, Fudan University Shanghai Cancer Center, Shanghai, China; 4 School of Medicine, Tongji University, Shanghai, China; 5 Center of Reproductive Medicine, Xinhua Hospital Affiliated Shanghai Jiao Tong University, School of Medicine, Shanghai, China; 6 Information Center, Shanghai Key Laboratory of Maternal Fetal Medicine, Shanghai Institute of Maternal-Fetal Medicine and Gynecologic Oncology, Shanghai First Maternity and Infant Hospital, School of Medicine, Tongji University, Shanghai, China

**Keywords:** infertility men, ICSI-ET, nomogram, risk factors, clinical pregnancy

## Abstract

**Objective:**

To identify risk factors influencing clinical pregnancy outcomes in infertile men undergoing intracytoplasmic sperm injection (lCSl) and to establish and validate a nomogram prediction model.

**Methods:**

A total of 1,037 infertile men who underwent lCSl-fresh embryo transfer (lCSl-ET) at the Reproductive Medicine Center of Tongji University Affiliated Obstetrics and Gynecology Hospital from February 2023 to February 2024 were included. Differences in demographic and laboratory indicators between 403 pregnancy cycles (study group) and 634 non-pregnancy cycles (control group) were analyzed. Lasso regression was applied to select predictive variables, which were further used in multivariate logistic regression to construct the nomogram. External validation was conducted using data from 290 infertile men who underwent ICSl treatment at Xinhua Hospital Affiliated to Shanghai Jiao Tong University School of Medicine (March-June 2024). Model performance was evaluated using the area under the receiver operating characteristic curve (AUC), confusion matrix and Decision Curve Analysis (DCA).

**Results:**

Age, body mass index, smoking, drinking, daily sleep time, daily exercise time, stress, progressive sperm motility rate, and sperm DNA fragmentation index were identified as predictors. In the training set, the model achieved an AUC of 0.919 (95% Cl: 0.900-0.938), the accuracy was 85.3% (95% CI:82.7%~87.5%), the sensitivity was 85.4% (95% CI: 81.1%~89.1%), the specificity was 85.2% (95% CI: 82.0%~88.1%). The validation set show that AUC of 0.930 (95% C1: 0.892-0.968), the accuracy was 80.7% (95% CI:74.8%~85.5%), the sensitivity was 78.8% (95% CI: 68.5%~86.9%), and the specificity was 81.9% (95% CI: 73.8%~88.4%). The external validation set results show that the AUC was 0.918 (95%CI: 0.876-0.959), the accuracy was 93.8% (95% CI: 90.4-96.0%), the sensitivity was 94.4% (95% CI: 88.4%~97.4%), and the specificity was 93.4% (95% CI: 88.8%~96.2%), indicating strong discrimination and calibration.

**Conclusion:**

Both sperm quality and lifestyle factors significantly affect clinical pregnancy outcomes in ICSl-ET cycles among infertile men. The developed nomogram demonstrates excellent predictive accuracy and reliability, providing a useful tool for clinicians to deliver individualized counseling and targeted interventions.

## 1 Introduction

The incidence rate of infertility shows a younger trend. By 2020, the incidence rate of infertility in China has reached 17.6%, of which the independent factors of men account for about 30%, and the common factors of men and women account for about 20% (1). The emergence of assisted reproductive technology (ART) has brought hope to infertile families. Although the success rate of *in vitro* fertilization/intracytoplasmic sperm injection embryo transfer (IVF/ICSI-ET) treatment has reached 40% to 50%, many infertile families still face the outcome of pregnancy failure. The clinical pregnancy outcomes of assisted reproduction are closely related to the quality of male sperm (2), and there is increasing evidence that lifestyle changes affect the fertility outcomes of assisted reproduction (3). Abnormal semen is the main cause of male infertility, and in addition, 30% to 50% of individuals with abnormal semen parameters cannot find a clear cause (4). Considering the high cost of IVF and the negative impact of treatment failure, it is essential to study the factors related to the outcomes of assisted reproduction in infertile men. Nomograms are one of the most common visualization forms of clinical prediction models that can predict the probability of outcome events based on patient characteristics (5) and are widely used in clinical settings. Therefore, this study aims to investigate the clinical data of infertile men undergoing assisted reproduction, analyze the main factors affecting their reproductive outcomes, and establish a nomogram prediction model for reproductive outcomes, in hopes of providing references for subsequent prognostic treatment and intervention.

## 2 Materials and methods

### 2.1 Research objects

From February 2023 to February 2024, infertile male patients who underwent ICSI-ET assisted reproduction at the Obstetrics and Gynecology Hospital affiliated with Tongji University were selected as the modeling group and internal verification group. Additionally, from March 2024 to June 2024, infertile males who received ICSI-ET assisted reproduction at the Reproductive Medicine Center affiliated with Shanghai Jiao Tong University School of Medicine’s Xinhua Hospital were chosen as the external verification group. All enrolled cases were consecutive and were divided into study and control groups based on whether clinical pregnancy occurred. Inclusion criteria: (1) The diagnostic criteria for infertile males in the “World Health Organization Human Semen Inspection and Processing Laboratory Manual (6th Edition)” (6); (2) Normal male reproductive system and physical examination, with no medical history affecting sperm quality (for example, diabetes, erectile dysfunction, etc.); (3) No treatment affecting sperm quality before semen examination; (4) Those who are informed and voluntarily participate in this study. Exclusion criteria: (1) Patients who received donor sperm, surgical sperm extraction, or frozen sperm assisted reproduction in this assisted reproduction cycle; (2) Those with severe chronic diseases, tumors, or other conditions; (3) Patients whose spouse has poor ovarian reserve function: baseline follicle-stimulating hormone ≥10mIU/mL or antral follicle count<5; uterine malformations, endometriosis, recurrent miscarriage patients; (4) Both partners have chromosomal or genetic abnormalities; (5) Patients without transferable embryo cycles, embryo accumulation, embryo freezing preservation, or those who have not completed embryo transfer.

The sample size was estimated according to the cross-sectional study sample size calculation formula (7).


$$\text{n}=\frac{Z^{2}\cdot P\cdot (1-P)}{\delta^{2}}$$

α=0.05, Z(1-α)/2 = 1.96; Previous surveys showed (8) P = 0.26, controlling the sampling error δ to 10% of the total rate, δ=0.05, calculated that the sample size is at least 296 cases, considering the 10% invalid response rate, the total sample size required is 326 cases.

This study was approved by the Ethics Committee of the Obstetrics and Gynecology Hospital affiliated with Tongji University (Ethical Number: KS2313).

### 2.2 Methods

#### 2.2.1 Investigation tools

(1) General Demographic Information: This was self-designed with reference to the “Chinese Expert Consensus on Male Fertility Assessment” (9), including age, body mass index (BMI), smoking history, alcohol drinking, coke drinking (>500ml/d), coffee drinking (500ml/d), hot spring (>1 time/week), working environment with high heat and radiation, daily sleep time, and daily exercise time. (2) Sperm Quality and Secretion Information: The semen analysis results of the researcher on the day of ICSI-ET assisted reproduction were collected. The researcher was advised to abstain from sexual activity 2~7 days before the test, using a unified method for semen and secretion processing and analysis, and extracting the researcher’s semen parameter information from the hospital HIS (Hospital Information System) system. (3) Serum Testosterone (T): Collect 3mL of fasting venous blood and determine the level using a chemiluminescence method after separating and obtaining the serum. (4) Self-Rating Depression Scale (SDS) and Self-Rating Anxiety Scale (SAS) (10): Based on the patient’s self-evaluation of symptoms within the last week, the total raw score is obtained by adding up the scores of the 20 items on the scale. Standard score = raw score × 1.25. According to the Chinese norm results, the standard score for SDS is 53 points, with 53~62 points indicating mild depression, 63~72 points indicating moderate depression, and >72 points indicating severe depression; the standard score for SAS is 50 points, with 50~59 points indicating mild anxiety, 60~69 points indicating moderate anxiety, and >70 points and above indicating severe anxiety. (5) Chinese Perceived Stress Scale (CPSS) (10): Developed by Cohen et al. in 1983 and revised by Yang Tingzhong et al. in 2003, the Cronbach’s alpha is 0.780, with high structural validity. The scale consists of 2 dimensions and 14 items, with total scores ranging from 11 to 26 indicating a lower level of perceived stress, 27 to 41 indicating moderate stress, and >42 indicating a high level of stress. (6) Athens Insomnia Scale (AIS) (11): Designed in 1985, the scale consists of 8 items, with total scores ranging from 0 to 24. Scores of 0 to 3 indicate no sleep disorders, 4 to 6 indicate suspected insomnia, and total scores >6 indicate insomnia. (7) Observation indicators of assisted reproduction outcomes: In this assisted reproduction cycle, if the serum HCG is >10 mIU/mL 14 days after fresh embryo transfer, it is diagnosed as HCG positive. Those with HCG positive status will undergo a B-ultrasound examination 2 weeks later to check for intrauterine pregnancy. The presence of a gestational sac and embryo is diagnosed as clinical pregnancy.

#### 2.2.2 Investigation methods

(1) Questionnaire Survey: After obtaining the consent of the patients, a questionnaire survey was conducted on the patients who met the inclusion and exclusion criteria by the male specialist nurse on the day of egg retrieval for ICSI. (2) Laboratory Data: Extracted from the clinical electronic medical record system. The questionnaire used a unified instruction asking patients to fill out the questionnaire based on them on the spot and provided sufficient time and an independent environment. The questionnaires were collected after checking for no missing items.

#### 2.2.3 Statistical methods

Data analysis was conducted using R4.2.2 software. Kolmogorov Smirnov test is used to test the normality distribution of the data. According to the distribution of the data, the metric data with normal distribution is represented by mean ± standard deviation ( $\bar{x}$± s); Non normally distributed metric data is represented by the median (M) [interquartile range, IQR]; Count is expressed in frequency (percentage) [n (%)]. Lasso regression was used to screen the feature variables, and the selected variables were included in a multi factor logistic regression to construct a nomogram prediction model. Among them, 80% were randomly sampled without replacement as the modeling group, and the remaining 20% were used as the internal validation group. Bootstrap resampling method (1000 times) was used for internal validation. Evaluate the performance of the model by plotting receiver operating characteristic (ROC) curves, Hosmer-Lemeshow test, Brier score, and DCA curves. The test level is 0.05, with P<0.05 indicating statistically significant differences.

For calibration assessment, the developed model was frozen, and its linear predictor (LP = X·β) was computed for each observation in the training, internal validation, and test sets. Logistic recalibration was then performed by fitting two models: the calibration slope was estimated from glm (outcome ~ LP, family = binomial), and the calibration-in-the-large (intercept) was estimated from glm (outcome ~ 1 + offset (LP), family = binomial). This process fits the recalibration framework logit(P) = a + b × LP, where a is the calibration intercept and b is the calibration slope. Wald 95% confidence intervals were computed for both statistics. It is noted that these were assessment fits only, and no model coefficients were updated.

It is worth noting that Lasso regression was used for variable screening before conducting multiple logistic regression in this study. Therefore, the estimated Odds Ratio and p-value in the final model may have post selection bias.

## 3 Results

### 3.1 Results of data collection

**Figure 1:**
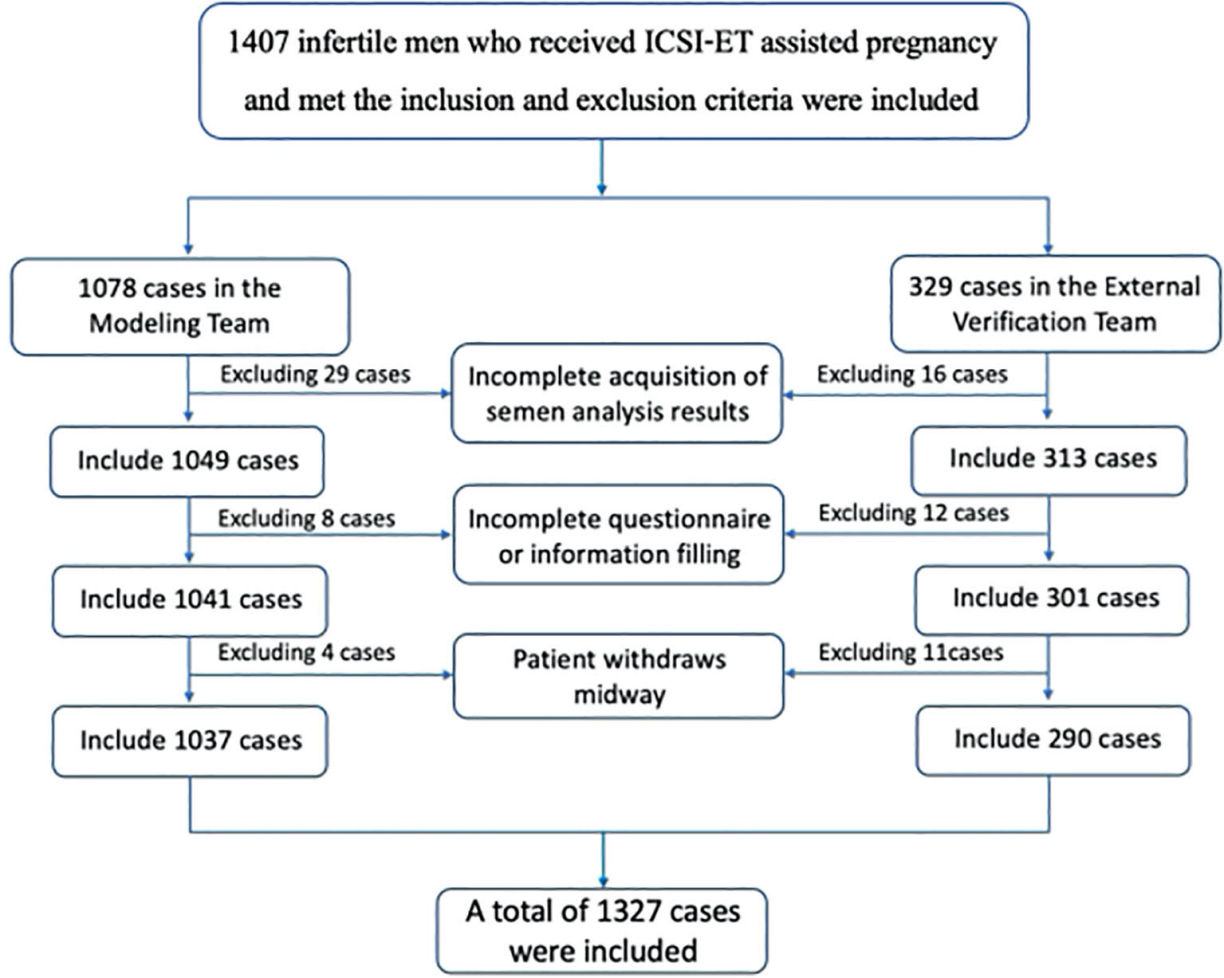
Process flow chart for subject inclusion.

The study included a total of 1407 ICSI-ET assisted pregnancy cycles that met the inclusion and exclusion criteria, of which 1078 were in the modeling group and 329 in the external validation group. 80 cycles with incomplete data collection were excluded, resulting in a final inclusion of 1037 cases in the modeling group and 290 cases in the external validation group, totaling 1327 cases. The inclusion flowchart is shown in **Figure 1**.

Within the modeling group, 403 cases (38.86%) of ICSI-ET cycles achieved clinical pregnancy, and there was no statistical difference in the general information of the female party in this cycle (P>0.05), as shown in **Table 1**.

**Table 1:** General information comparison of women receiving assisted conception in this cycle (n=1037).

Item	Research group (*n* = 403)	Control group (*n* = 634)	*t*/χ^2^	*P* values
Age (year)	33.25 ± 2.93	33.44 ± 2.81	-1.044	0.297
BMI (kg/m^2^)	22.26 ± 1.53	22.37 ± 1.48	-1.151	0.250
Infertility Duration(year)	2.84 ± 0.81	2.93 ± 0.96	-1.562	0.119
Assisted Pregnancy Program			2.891	0.235
Long case of early follicular phase	71(17.62%)	138(21.77%)		
Long term plan	83(20.59%)	117(18.45%)		
Antagonist regimen	249(61.79%)	379(59.78%)		
Endometrial thickness on the day of transplantation(mm)	9.79 ± 1.08	9.67 ± 1.14	1.686	0.092
Number of high-quality embryos	3.82 ± 0.54	3.78 ± 0.49	1.231	0.219
Type of embryo transfer			0.094	0.759
Cleavage stage	388(96.28%)	615(96.69%)		
Blastocyst stage	15(3.72%)	21(3.31%)		

### 3.2 Clinical characteristics of infertile men in modeling group

This study included a total of 1037 subjects who met the inclusion and exclusion criteria. Among the subjects, the youngest was 22 years old and the oldest was 49 years old, with an average age of (35 ± 5.35) years old; Among them, a total of 403 cases (38.86%) achieved clinical pregnancy. The specific distribution of information is shown in **Tables 2**, **3**.

**Table 2:** Distribution of clinical data of infertile men in the modeling group (*n* = 1037).

Item	Group	Cases	Proportion (%)
Age (year)	20∼25	4	0.39
	26∼35	611	58.92
	36∼45	370	35.68
	>45	52	5.01
BMI(kg/m^2^)	≤18.5	8	0.77
	18.6∼23.9	313	30.18
	24.0~27.9	489	47.16
	≥28.0	227	21.89
High heat and high radiation working environment	Yes	49	4.73
	No	988	95.27
Smoking (cigarettes/d)	None	472	45.52
	0~10	154	14.85
	11~20	177	17.07
	>20	234	22.56
Alcohol drinking (mL/d)	None	527	50.82
	0~50	191	18.42
	51~100	149	14.37
	>100	170	16.39
Coke drinking	Yes	202	19.48
	No	835	80.52
Coffee drinking	Yes	244	23.53
	No	793	76.47
Hot Spring	Yes	66	6.36
	No	971	93.64
Daily sleep time (h/d)	<7	444	42.82
	7~9	546	52.65
	>9	47	4.53
Daily Exercise Time(h/d)	Almost none	263	25.36
	0~0.5	361	34.81
	0.5~1	270	26.04
	>1	143	13.79
Anxiety	None	643	62.01
	Mild	231	22.27
	Moderate	105	10.13
	Severe	58	5.59
Depression	None	630	60.75
	Mild	308	29.70
	Moderate	72	6.95
	Severe	27	2.60
Stress	None	259	24.98
	Moderate pressure	455	43.88
	High pressure	290	27.96
	Heavy pressure	33	3.18
Insomnia	None	231	22.28
	Suspected Insomnia	406	39.15
	Insomnia	400	38.57

**Table 3:** Distribution of laboratory data for infertile men in the model group (*n* = 1037).

Item	$\bar{x}$ ± *s*	Classification	Min	Max
Testosterone	5.53 ± 1.95		2.01	12.90
Progressive Motility Sperm Rate (%)	22.66 ± 6.06	/	3.40	31.9
Non-Progressive Motility Sperm Rate (%)	19.82 ± 5.19	/	5.80	44.0
Immotile Sperm Rate (%)	56.18 ± 8.54	/	25.80	79.80
Head deformity rate (%)	79.43 ± 3.85	/	67.00	90.00
Mixed malformation rate (%)	17.95 ± 3.71	/	6.00	30.00
Normal Form rate (%)	2.70 ± 0.941	/	0.00	5.00
Sperm DFI (%)	27.92 ± 6.77	/	14.64	64.81
Sperm Viability Rate (%)	48.62 ± 7.99	/	10.00	66.00
Sperm Concentration (10^6^/mL)	11.08 ± 6.60	/	2.00	62.70
Semen volume (ml)	3.50 ± 1.46	/	0.30	11.50
Semen PH	7.29 ± 0.19	/	7.00	8.00
Mycoplasma	/	Negative	910	87.75%
	/	Positive	127	12.25%
Chlamydia	/	Negative	908	87.56%
	/	Positive	129	12.44%
Anti-sperm antibody	/	Negative	933	89.97%
	/	Positive	104	10.03%

### 3.3 Screening of risk factors for clinical pregnancy outcomes of ICSI-ET in infertile men

**Figure 2:**
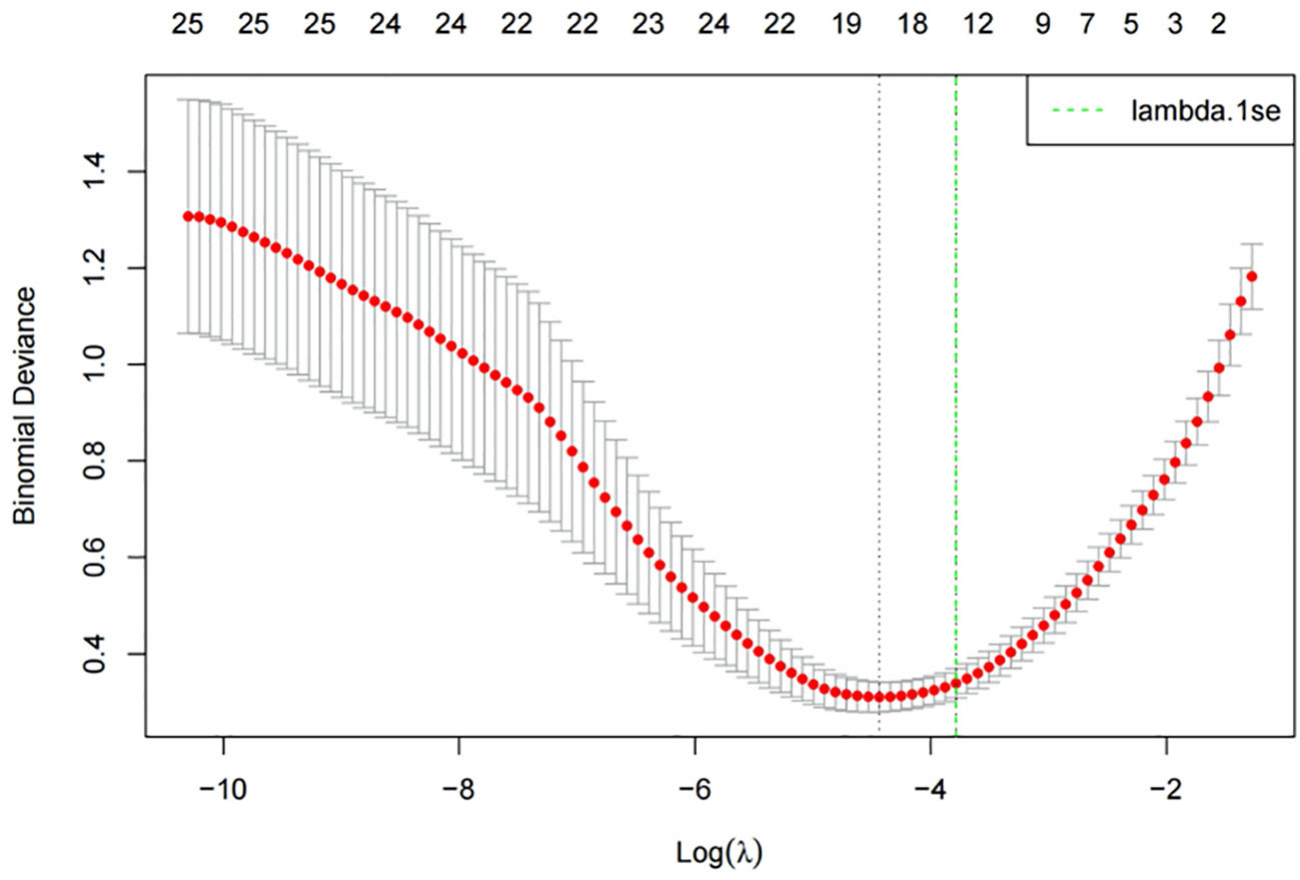
Lasso regression cross-curve validation diagram.

**Figure 3:**
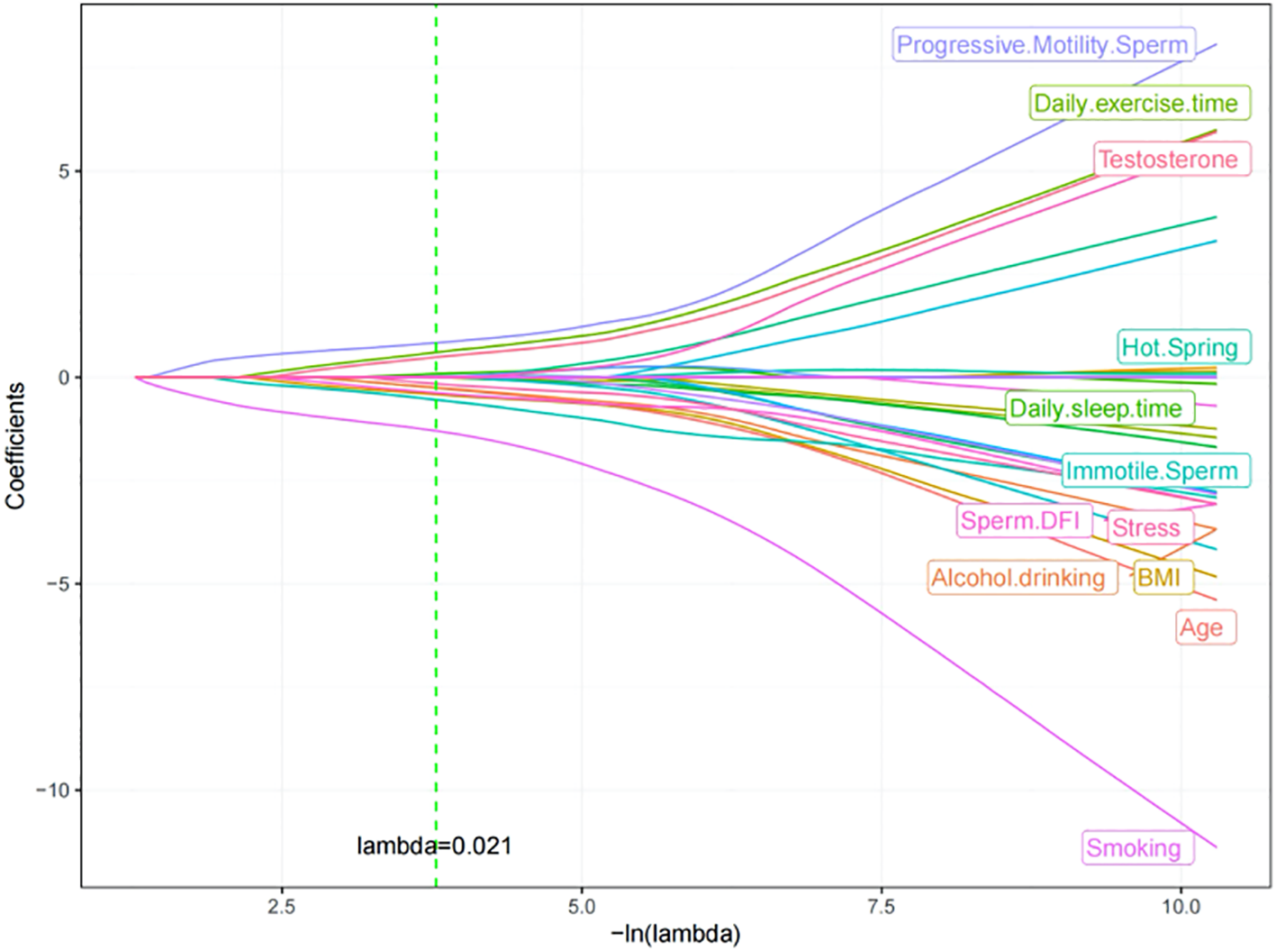
Lasso regression coefficient distribution trajectory map.

LASSO regression was performed using 30 independent variables of clinical and laboratory data, with ICSI-ET assisted pregnancy in infertile men as the dependent variable (No=0, Yes=1). Using a 10-fold crossover method, the model was validated by fitting different combinations of variables. According to the lambda. 1 se variable screening criterion, the log (λ)=0.021 model had excellent performance and was simplified. A total of 12 risk factors were screened, including: Progressive Motility Sperm Rate, Daily Exercise Time, Testosterone, Hot Spring, Daily sleep time, Immotile Sperm Rate, Sperm DFI, Stress, Alcohol drinking, BMI, Age, Smoking. See **Figures 2**, **3** for details.

### 3.4 Multiple logistic regression analysis of clinical pregnancies in infertile men undergoing ICSI-ET

Using whether clinical pregnancy was achieved in the ICSI-ET cycle of infertile men as the dependent variable (no=0, yes=1), a multiple logistic regression analysis was conducted with the 12 predictors screened by Lasso regression as independent variables. The assignment methods for independent variables are: smoking: 0=no, 1 = 0~10 cigarettes, 2 = 11~20 cigarettes, 3= >20 cigarettes; drinking: 0=no, 1 = 0~50 mL/d, 2 = 51~100mL/d, 3= >100 mL/d;daily sleep time: 0=<7h, 2 = 7-9h, 3=> 9h; daily exercise time: 0=almost none, 1 = 0~0.5h, 2 = 0.5-1h, 3=> 1h; Hot Spring: 0=Yes, 1=No; stress: 0=none, 1=moderate, 2=high, 3=severe stress. The final predictor variable of this study is the variable that has statistical significance after multiple logistic regression (p<0.05). The final predictor variables are Age, BMI, whether to smoke, whether to drink alcohol, daily sleep time, daily exercise time, Progressive Motility Sperm rate, sperm DFI, stress. The ORs/p-values presented are estimated by model selection data and should be considered as predictive weights rather than accurate performance estimates. The validity of their statistical inferences is affected by selection bias. Therefore, these values are mainly used to evaluate the predictive ability of nomogram models rather than informal causal inference. The results are detailed in **Table 4**.

**Table 4:** Multiple logistic regression analysis results of clinical pregnancy outcomes of ICSI-ET in infertile men (*n* = 1037).

Research factor	B	S.E.	*P*	*OR*	95% *CI*
Constant	-17.825	1.960	0.000		
Age	-0.088	0.021	0.000	0.916	0.878~0.955
BMI	-0.169	0.036	0.000	0.844	0.787~0.905
Whether to smoke (with No as reference)			0.000		
0~10 cigarettes/d	-1.582	0.314	0.000	0.206	0.111~0.380
11~20 cigarettes/d	-1.834	0.317	0.000	0.160	0.086~0.298
>20 cigarettes/d	-2.272	0.316	0.000	0.103	0.056~0.191
Whether to drink alcohol (with No as reference)			0.002		
0~50 mL/d	-1.133	0.323	0.000	0.322	0.171~0.607
51~100 mL/d	-0.379	0.305	0.215	0.685	0.376~1.246
>100 mL/d	-0.700	0.331	0.035	0.497	0.259~0.951
Daily sleep time (with< 7h as reference)			0.000		
7~9h/d	1.008	0.228	0.000	2.740	1.754~4.280
> 9h/d	1.436	0.566	0.011	4.204	1.385~12.759
Daily exercise time (with Almost none as reference)			0.000		
0~0.5h/d	0.599	0.290	0.039	1.820	1.031~3.214
0.5~1h/d	0.998	0.310	0.001	2.712	1.477~4.982
>1h/d	1.488	0.351	0.000	4.428	2.224~8.814
Progressive Motility Sperm Rate (per 10 percentage points)	0.126	0.022	0.000	1.134	1.086~1.185
Sperm DFI (per 10 percentage points)	-0.126	0.018	0.000	0.882	0.851~0.914
Immotile Sperm Rate (per 10 percentage points)	0.009	0.013	0.488	1.009	0.983~1.037
Testosterone	-0.037	0.054	0.495	0.964	0.868~1.071
Hot Spring (with YES as reference)	-0.135	0.437	0.758	0.874	0.371~2.059
Stress (with None as reference)			0.036		
Moderate pressure	-0.264	0.251	0.294	0.768	0.469~1.257
High pressure	-0.219	0.371	0.555	0.804	0.388~1.662
Heavy pressure	-1.533	0.598	0.010	0.216	0.067~0.697

Nagelkerke *R*^2^ = 0.722.

### 3.5 Construction of nomogram and evaluation of model efficiency

**Figure 4:**
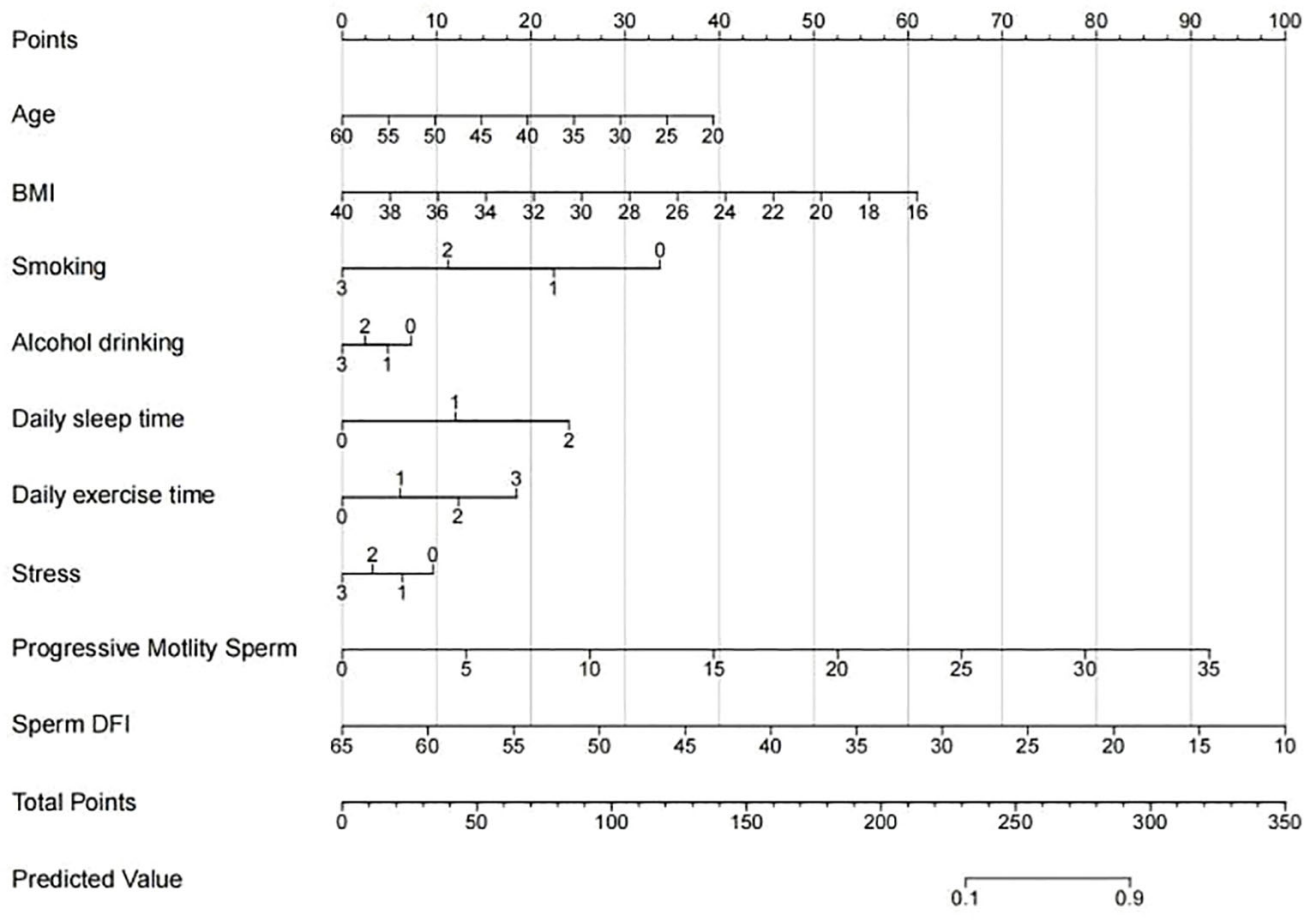
Nomogram of whether men with infertility achieved clinical pregnancy through ICSI-ET assistance.

**Figure 5:**
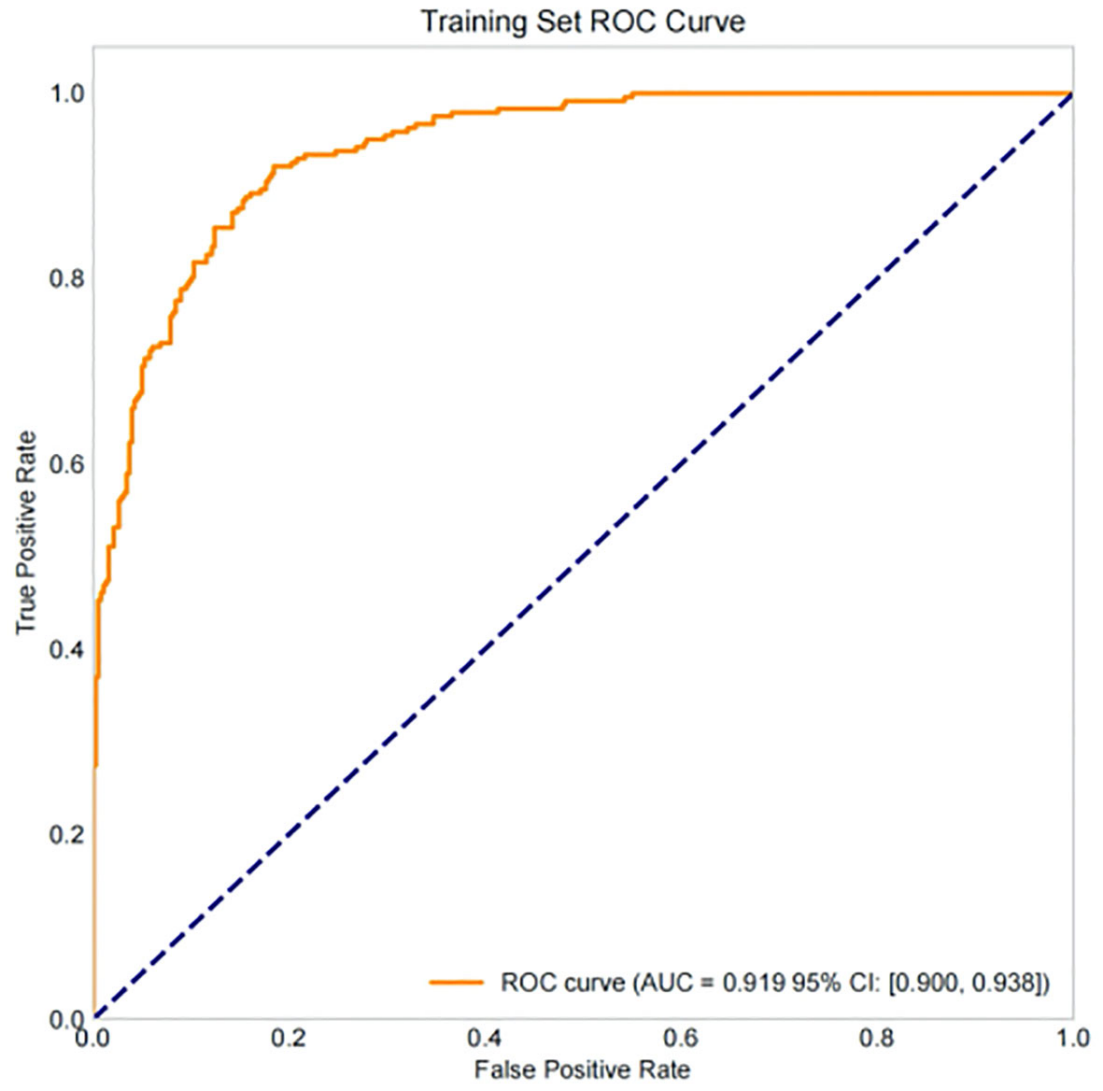
Analysis of ROC curve of training set.

**Figure 6:**
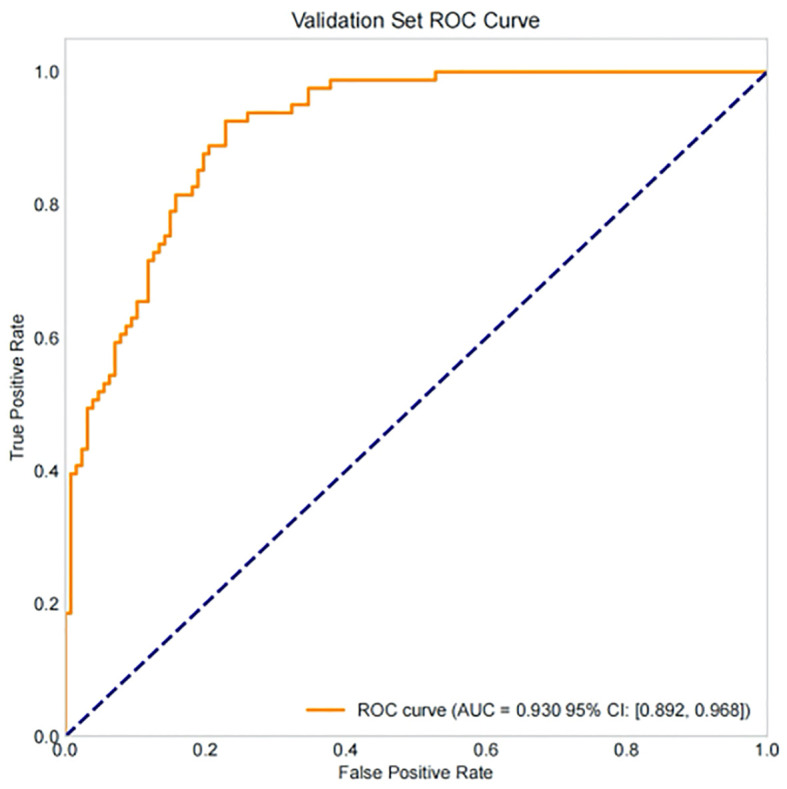
ROC curve analysis of the validation set.

**Figure 7:**
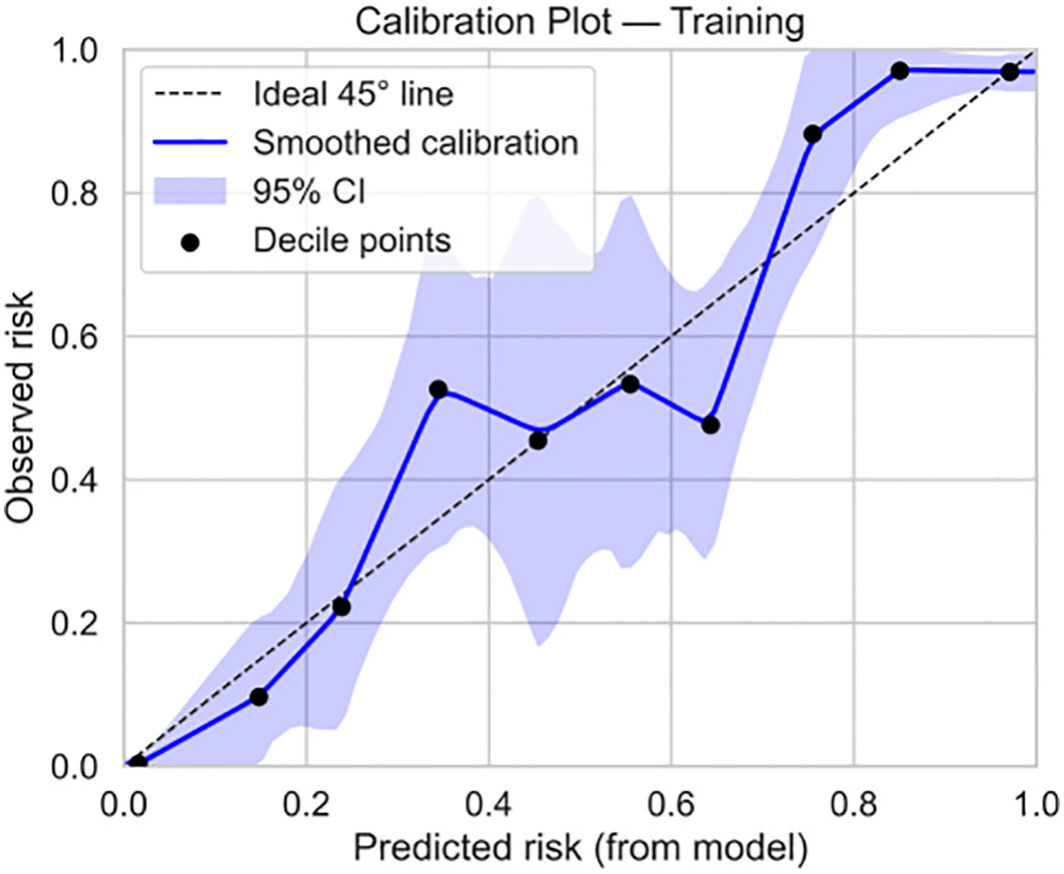
Calibration curve for training set.

**Figure 8:**
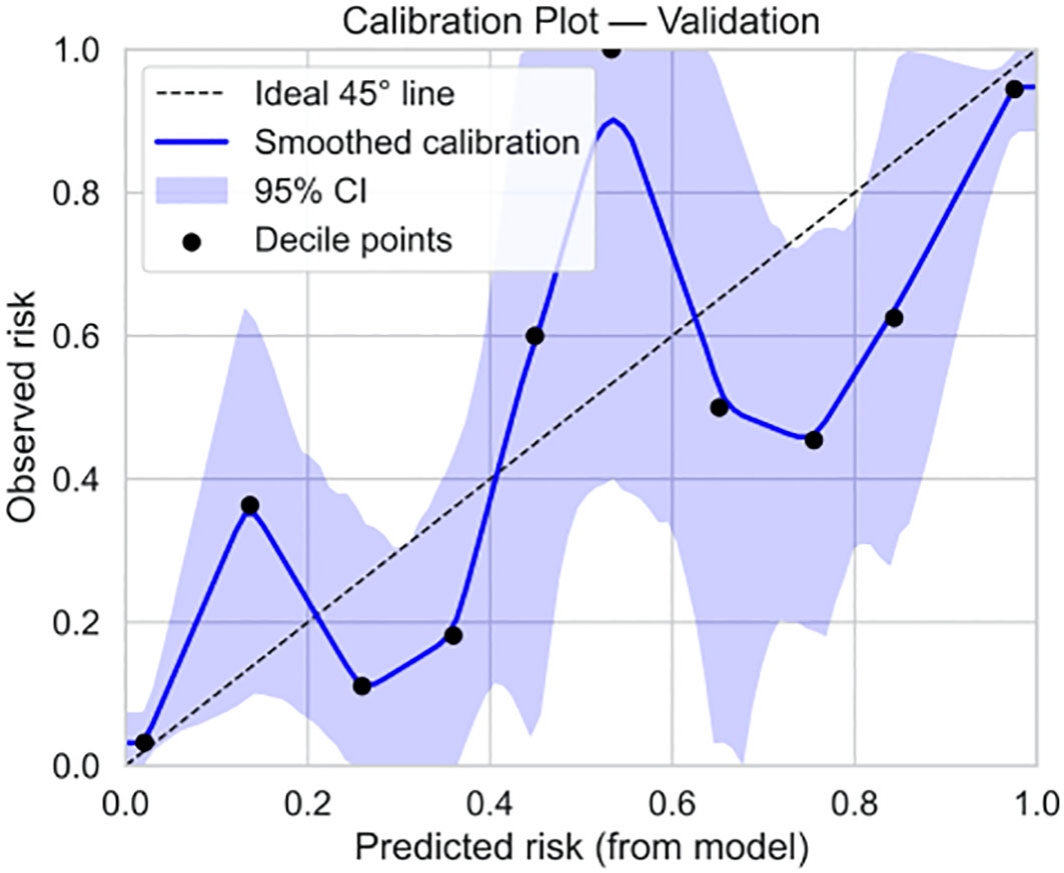
Validation set calibration curve.

Based on the 9 independent predictive factors screened by lasso regression, a nomogram prediction model for clinical pregnancy in infertile male patients undergoing ICSI-ET was constructed, as shown in **Figure 4**. The results showed that in the training set, AUC of 0.919 (95%CI: 0.900~0.938), Hosmer-Lemeshow *P* = 0.957, and Brier Score of 0.073. In the validation set, AUC was 0.930 (95%CI: 0.892~0.968), Hosmer-Lemeshow *P* = 0.784, and Brier Score of 0.069, as shown in **Figures 5**–**8**, and **Table 5**.

**Table 5:** Detailed performance indicators of the model in each dataset.

Dataset	Case	TP	FP	TN	FN	Accuracy (95%CI)	Sens. (95%CI)	Spec. (95%CI)	PPV (95%CI)	NPV (95%CI)	Birer	Slope (95%CI)	Intercept (95%CI)
Training	829	271	75	432	51	85.3% (82.7~87.5%)	85.4% (81.2~88.9%)	85.2% (81.9~99.0%)	78.6% (74.0~82.6%)	90.2% (87.2~92.5%)	0.073	1.209 (0.984~1.433)	0.001 (-0.314~0.315)
Validation	208	61	23	104	20	80.7% (74.9~85.5%)	78.8% (68.8~86.3%)	81.9% (74.3~87.6%)	73.3% (63.1~81.5%)	86.0% (78.6~91.0%)	0.069	0.749 (0.546~0.953)	-0.380 (-0.905~0.145)
External validation	290	102	12	170	6	93.8% (90.4~96.0%)	94.4% (84.4~97.4%)	93.4% (88.8~96.2%)	89.5% (82.5~93.9%)	96.6% (92.8~98.4%)	0.077	1.113 (0.776~1.450)	-0.270 (-0.801~0.260)

**Figure 9:**
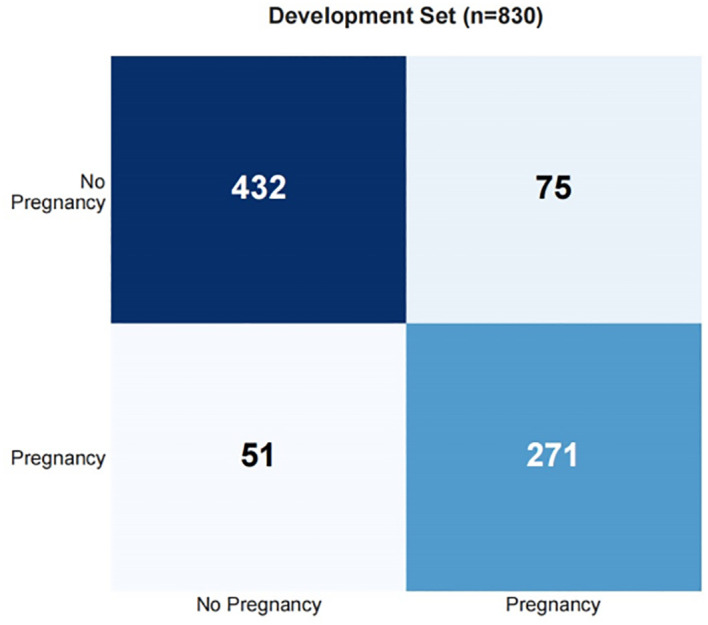
Confusion matrix for training set.

**Figure 10:**
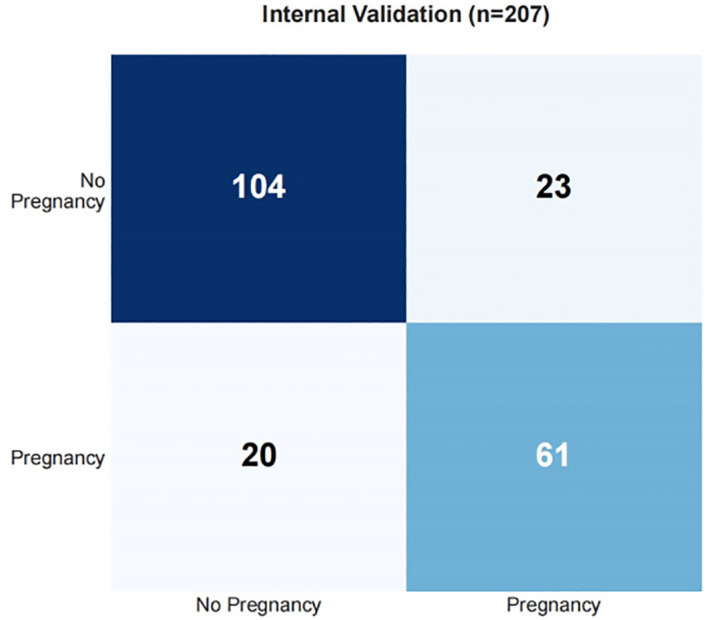
Confusion matrix for validation set.

Draw a confusion matrix to evaluate the sensitivity and accuracy of the model. A single operating threshold was pre-specified prior to validation. This threshold was determined by maximizing Youden’s J index (Sensitivity + Specificity - 1) on the development set alone. The optimal threshold was identified as 0.42. This pre-specified threshold of 0.42 was then locked and applied without any further adjustment to both the internal and external validation sets. The results showed that in training set, the accuracy was 85.3% (95% CI:82.7%~87.5%), the sensitivity was 85.4% (95% CI: 81.1%~89.1%), the specificity was 85.2% (95% CI: 82.0%~88.1%), in the internal validation set, the accuracy was 80.7% (95% CI:74.8%~85.5%), the sensitivity was 78.8% (95% CI: 68.5%~86.9%), and the specificity was 81.9% (95% CI: 73.8%~88.4%). See **Table 5**, **Figures 9**, **10**.

**Figure 11:**
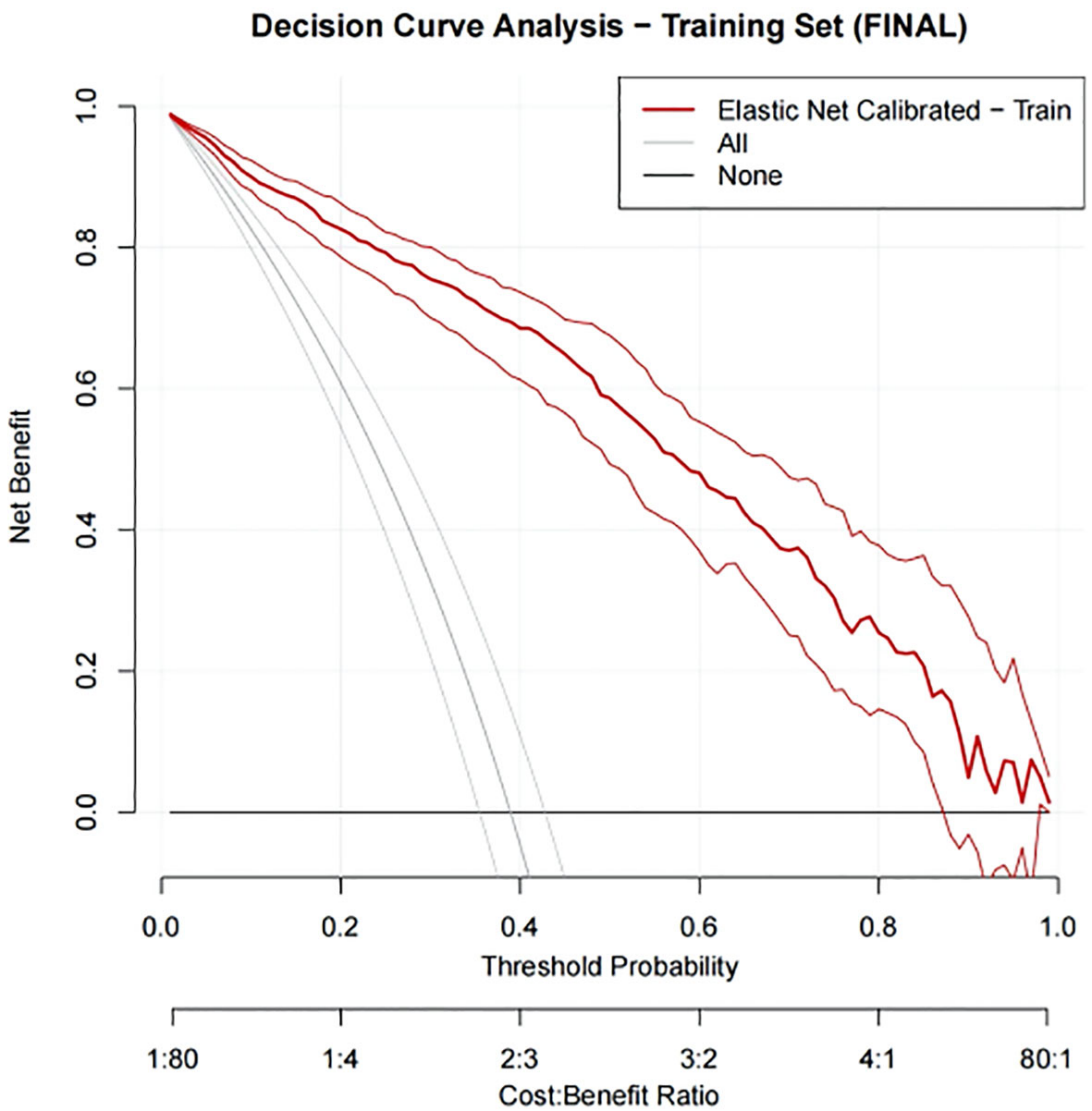
DCA curve for training set.

**Figure 12:**
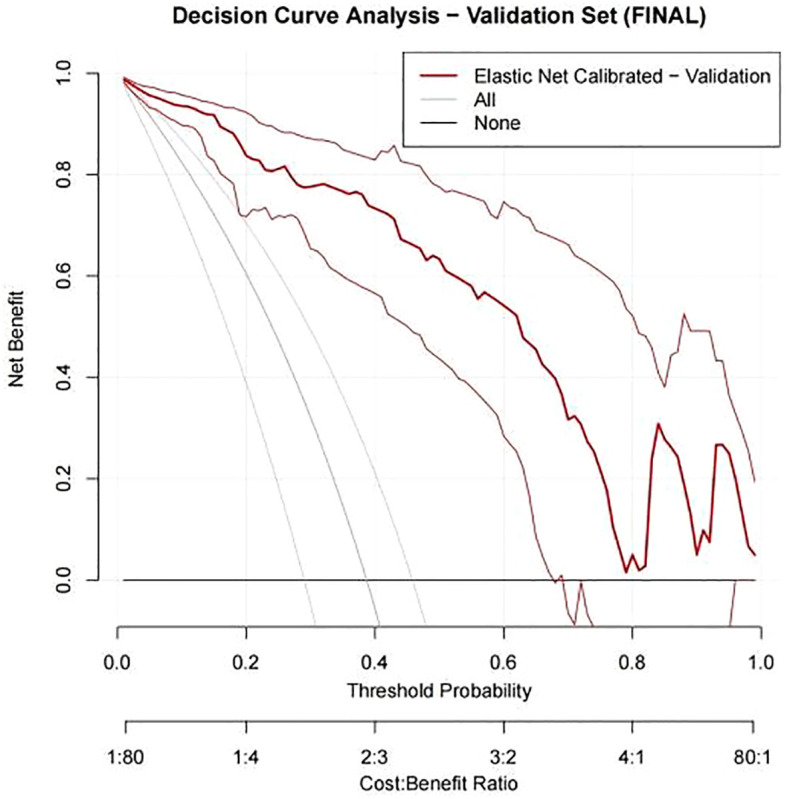
DCA curve for validation set.

In our study, DCA was used to evaluate the net benefit of using predictive models for clinical decision-making compared to models that were not applicable. The x-axis represents the threshold probability, which is the probability that the predictive model determines that infertile men will achieve clinical pregnancy; The y-axis represents the net benefit, which is the comprehensive benefit of assisted reproductive outcomes for infertile men under different strategies. In the training set, the net benefit curve of our nomogram model is significantly higher than the default strategies of “all treatment” and “no treatment” (**Figure 11**). Specifically, the model provides superior net benefits within a wide threshold probability range of approximately 0% to 81%. This indicates that within this risk tolerance range, using column charts to assist clinical decision-making for ICSI-ET pregnancy is beneficial. Similarly, in the internal validation set, the net profit curve of the model outperformed the default strategy within a threshold probability range of approximately 0% to 78% (**Figure 12**).

### 3.6 External validation of the nomogram

**Figure 13:**
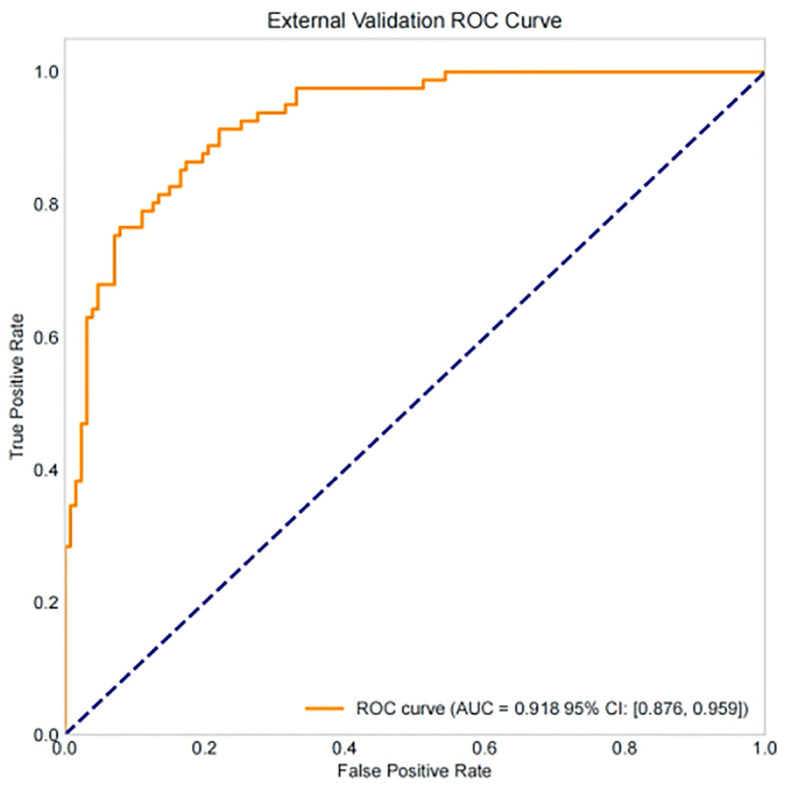
ROC curve of external verification set.

**Figure 14:**
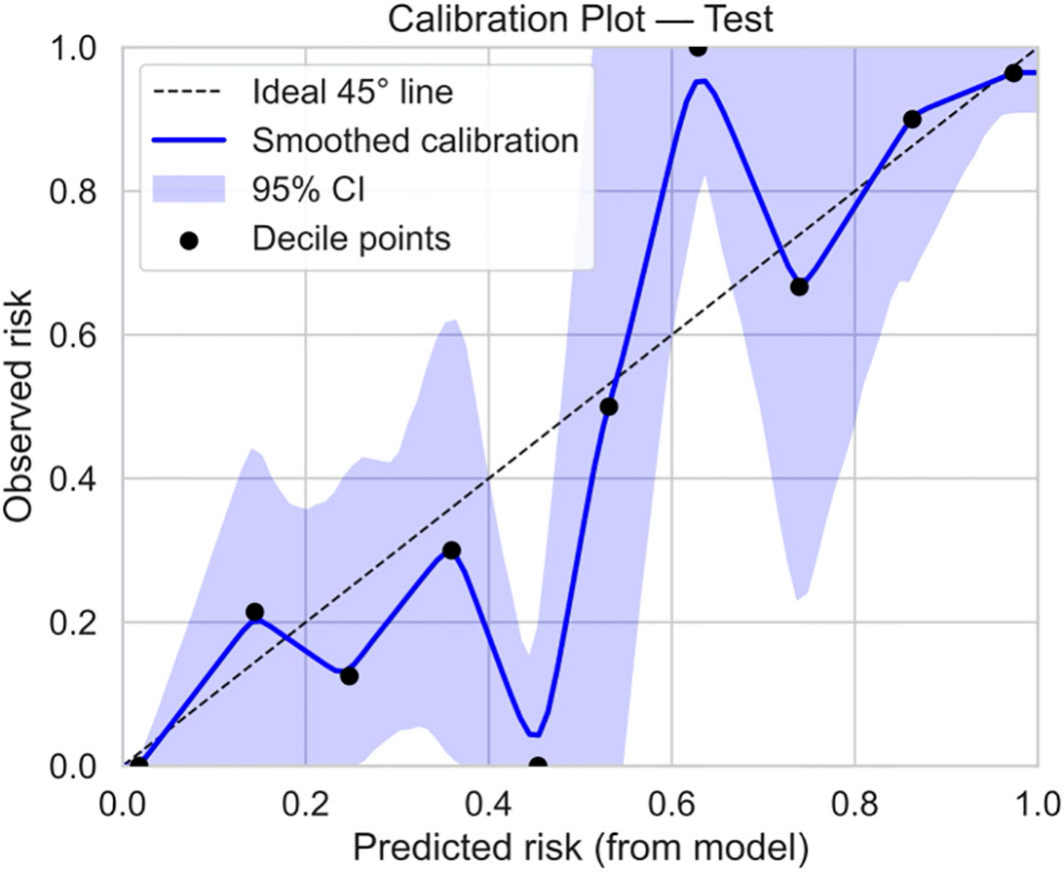
Calibration curves for the external validation set.

**Figure 15:**
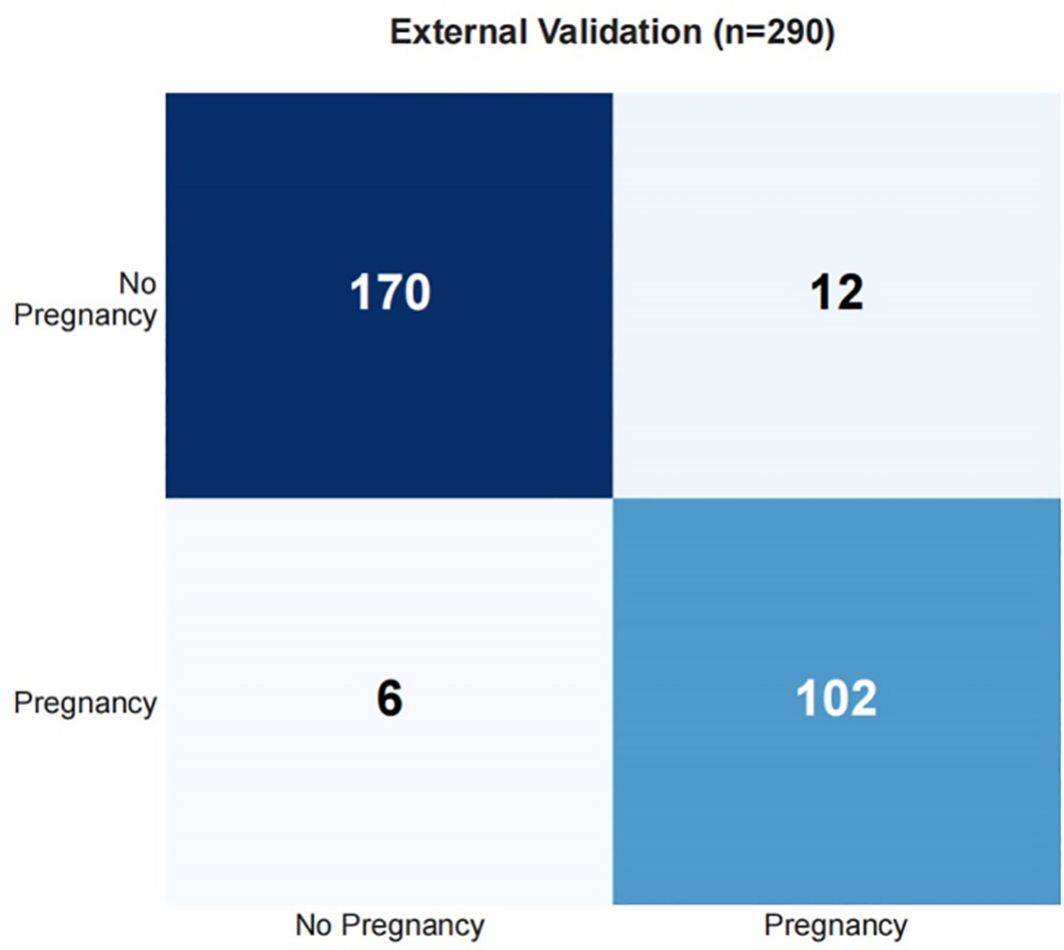
Confusion matrix for external validation set.

**Figure 16:**
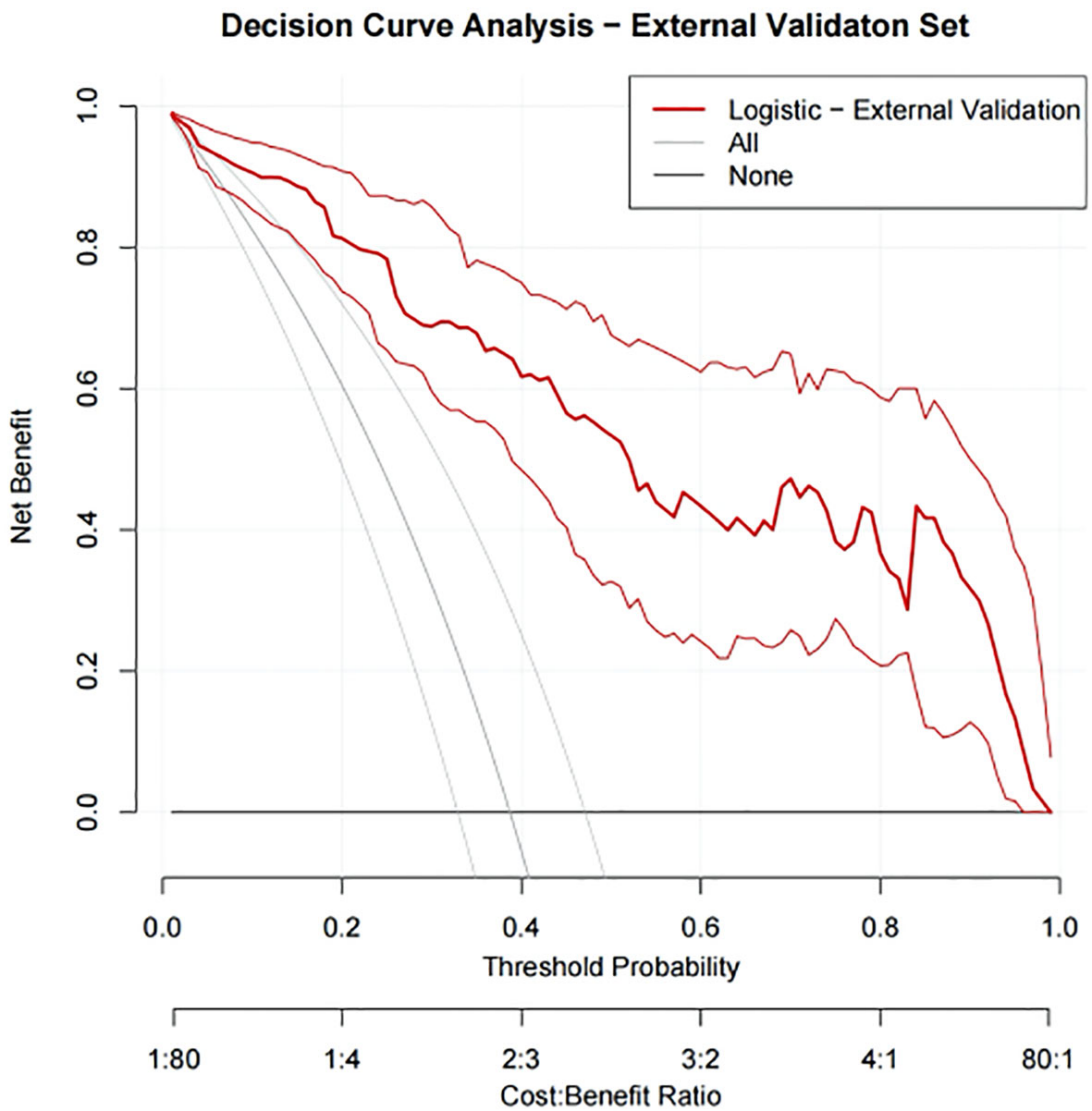
DCA curves for the external validation set.

Among 290 ICSI-ET cycles of the external validation group, 106 (36.55%) infertile men achieved clinical pregnancy. The constructed nomogram is directly applied to an external validation queue without the need for retraining or modifying the model coefficients. To ensure fairness and unbiased proof, using the same frozen model and preprocessing steps, all data steps are strictly replicated on the validation set without recalibrating the intercept and/or slope of the model. The external validation set results show that: the AUC was 0.918 (95%CI: 0.876-0.959), Hosmer-Lemeshow *P* = 0.717, and Brier Score was 0.077, see **Figures 13**, **14**, and **Table 5**. The confusion matrix of the external validation group showed that the accuracy was 93.8% (95% CI: 90.4-96.0%), the sensitivity was 94.4% (95% CI: 88.4%~97.4%), and the specificity was 93.4% (95% CI: 88.8%~96.2%), the PPV was 89.5% (95%CI: 82.5%~93.9%), and the NPV was 96.6% (95%CI: 92.8%~98.4%), see **Figure 15** and **Table 5**. In the external validation set, the nomogram demonstrated a positive net benefit over the default strategies for threshold probabilities ranging from 0% to 83%, as shown in **Figure 16**.

## 4 Discussion

### 4.1 Sperm quality is a key factor in the success of ART treatment

The factors affecting infertility are numerous and the mechanism of occurrence is complex. A comprehensive understanding of the mechanism and symptoms of infertility is crucial for formulating individualized treatment plans. The three core indicators revealed in this study, namely DFI, Progressive Motility Sperm Rate, essentially reflect the multi-level biological status of sperm from genetic integrity to functional execution. Sperm nuclear DNA is a carrier of genetic information and an essential guarantee for the fertilization process, greatly influencing the success rate of pregnancy and the healthy development of offspring. By detecting the integrity of sperm nuclear DNA, one can reveal the function of sperm at the molecular level and assess the fertility of patients. An elevated DFI is not only positively correlated with oxidative damage markers such as 8-hydroxydeoxyguanosine (8-OHdG) (12) but may also induce epigenetic modification disorders through abnormal histone-protamine replacement (13). Yang H et al. (14) showed that defects in the integrity of sperm genetic material may influence an increase in sperm DFI, various sperm parameters change accordingly and are indirectly associated with the failure of assisted reproductive technology. In China, some scholars (15) combined DFI with sperm survival rate to predict the assisted reproductive pregnancy outcome (miscarriage rate) for infertile couples. The results showed that the AUC of DFI combined with sperm survival rate in predicting miscarriage rate in assisted reproduction patients was 0.863 (95%CI:0.832~0.937), demonstrating good predictive ability. The total number of forward motility sperm is one of the key factors for the success rate of pregnancy before assisted reproduction. The activity and quality of sperm can compensate for the defects in sperm morphology (16) and with age, sperm quality will decline, it may be related to the decreased fertilization ability of sperm and may be associated with pregnancy outcomes to some extent; Genetic variations in male reproductive cells may accumulate with age and have some correlation with the development of fertilized eggs and the outcome of pregnancy (17). Youn et al. (18) believe that combining sperm motility, sperm morphology, and other parameters has significant prognostic value for assisted reproductive outcomes; Unlike this study, Luo et al. (19) predicted the prognostic factors of pregnancy outcomes for intrauterine insemination with multiple logistic regression models. Sperm motility and sperm morphology rate were identified as predictable prognostic factors for the pregnancy outcomes of artificial insemination. In this study, the impact of sperm morphology rate on assisted reproductive outcomes was not significant, which may be related to the different subjects included in the study.

### 4.2 The impact of unhealthy lifestyles on the fertility outcomes of infertile men

A poor lifestyle mainly damages sperm quality by increasing the level of oxidative stress in the body, producing free radicals (18), which may influence to negative outcomes in assisted reproductive technology. As a heavy consumer of cigarettes, the adult smoking rate in China reached as high as 27.7% in 2018, and the proportion of Chinese women exposed to passive smoking is even higher (20). The harm of passive smoking is even greater than that of active smoking, because the concentration of toxic substances in the sidestream smoke inhaled by passive smokers is higher compared to mainstream smoke. Long-term smoking increases the estradiol content in men, reduces the total testosterone content, affects semen parameters; nicotine can cause testicular and vas deferens atrophy, disrupt testicular and epididymal circulation and internal environmental material exchange, affect testicular sperm production, and reduce sperm concentration (21); in addition, smoking may also pass on mutated genes to descendants through epigenetics. A study identified 141 significantly differentially methylated CpGs associated with smoking, suggesting that smoking-induced heteromethylation of CpG islands in sperm DNA may affect the healthy development of embryos (22). Ethanol may have some negative effects on the male testicular spermatogenesis, which may be related to regression of the gonads and even testicular atrophy in severe cases. Long-term or excessive drinking can also cause gonadal poisoning, resulting in gonadal regression, low testosterone, abnormal gonadotropin, and even testicular atrophy in severe cases, affecting male fertility (23). Moreover, lack of exercise will also affect the outcome of assisted reproduction. Sitting for long periods may disrupt the temperature regulation of the testes and increase pressure on them, generate oxidative stress (24). Negative emotions can disrupt the level of reproductive hormones through the hypothalamus-pituitary-adrenal axis and sympathetic nervous system mediation, and can change the levels of inflammatory factors, causing a low-grade chronic inflammatory state, affecting the function of the reproductive immune system (25), reducing the success rate of ART conception. Sleep disorders are a frequent concomitant symptom in IVF patients with obvious psychological problems (26). Insufficient sleep reduces the secretion activity of male fertility hormones. Sleep disorders may influence sperm concentration, total number and testicular volume, and will also affect sperm morphology (27), which will also affect the outcome of ART assisted reproduction.

### 4.3 Age and BMI affect the assisted reproductive outcomes of infertile men

At present, there is still controversy over the impact of elderly men on the outcome of assisted reproductive pregnancy. In this study, the older the age, the lower the clinical pregnancy rate of assisted reproductive outcomes. According to a meta-analysis (28), Chinese men aged 40 years or older who receive IVF-ET treatment have a reduced incidence of fresh embryo transfer pregnancies, including biochemical and clinical pregnancies, which is consistent with the results of this study. As men age, the quality of their sperm may decrease, including sperm quantity, morphology, and vitality, which may lead to a decrease in sperm fertility and thus affect the outcome of pregnancy. Genetic variations in male reproductive cells may accumulate with age, which may increase the risk of chromosomal abnormalities or gene mutations, thereby affecting the development of fertilized eggs and the outcome of pregnancy; However, according to studies from other countries and regions (29), no correlation was found between age and pregnancy rate, which may be related to the characteristics of different races and populations.

In our study, BMI index was negatively correlated with assisted pregnancy outcomes. Studies (30) have shown that obesity can alter the secretion function of the hypothalamic pituitary gonadotropin axis, leading to a decrease in levels of gonadotropin-releasing hormone. BMI is positively correlated with luteinizing hormone and serum leptin, and negatively correlated with testosterone; Luteinizing hormone and follicle stimulating hormone are negatively correlated with sperm motility, while leptin can stimulate increased release of luteinizing hormone and follicle stimulating hormone; Obese patients have higher levels of estradiol and lower levels of testosterone, and testosterone levels are positively correlated with sperm motility; The decrease in testosterone may mainly lead to a decrease in sperm motility by affecting the ability to remove zinc from the epididymis. As BMI increases, the level of reactive oxygen species in semen increases, which may lead to membrane lipid peroxidation and decreased sperm motility; Obese patients’ sperm mitochondrial complexes are disrupted, releasing reactive oxygen species that affect the electron transport chain, reduce ATP synthesis, ultimately decrease sperm motility, and affect fertility outcomes (31). In addition, being overweight or obese can create an unfavorable environment for sperm production, such as high local body temperature in the external genitalia, which can reduce the ability of the testes to produce sperm and lead to the occurrence of asthenozoospermia (32).

### 4.4 The construction of a nomogram for the outcome of assisted reproduction in infertile men has clinical guiding significance

Our study constructed a nomogram model for the outcome of ICSI-ET assisted pregnancy in infertile men using 9 risk factors, including general patient information, semen quality, and lifestyle. We used the nomogram to assign scores to each influencing factor, and when the total score was between 230 and 280, the predictive probability was between 10% and 90%. In our results, the factor with the highest proportion of predictions for assisted reproductive outcomes is semen quality, which is consistent with previous studies; in addition, advanced age and obesity also have a negative impact on the predictive efficacy of assisted reproductive outcomes; meanwhile, smoking, alcohol consumption, lack of exercise, insomnia, and high stress remain the main risk factors affecting the fertility outcomes of this population, which deserves our attention. The nomogram model constructed by these 9 factors has significant predictive value and clinical application efficiency and can directly obtain the specific probability value of whether infertile men who receive ICSI will achieve clinical pregnancy through this model. This helps medical personnel make clinical decisions, provide a basis for comprehensive evaluation of semen quality and lifestyle of infertile populations, take active intervention measures to improve clinical outcomes, and develop personalized medical plans, thus having certain clinical significance.

The predictive model construction of this study embodies the deep integration of methodology and clinical thinking. In the variable screening stage, the application of LASSO regression effectively solves the problem of multicollinearity commonly found in traditional logistic regression, Through 10-fold cross validation, according to the selection criteria of lambda. 1 se variables, the model performs well and is simplified when log (λ)=0.021. At present, there are few reports on the research of the predictive model for predicting the outcome of assisted reproduction in male infertility patients using nomogram. In this study, we integrated multimodal data to analyze the daily exercise time, standardized psychological scale, and laboratory semen parameters in combination, further logistic regression was conducted using lasso regression to identify the variable combination with the strongest predictive power. Finally, multiple logistic regression was used to determine the final predictive factors and obtain the variable combination with the strongest joint predictive effect. The coefficients from the logistic regression model represent the predictive weights in the final model formula. These coefficients should not be used for formal statistical inference but should be understood as predictive weights used to evaluate the relative contribution of predictive factors to the predictive performance of the model. We emphasize that the focus of this study is to construct predictive models rather than unbiased causal inference.

Our study comprehensively validated the established clinical prediction model through ROC curve, calibration curve, and clinical decision curve analysis. The results showed that our model has strong predictive ability for ICSI-ET assisted pregnancy outcomes in infertile men, and the consistency between predicted risk and actual risk is good. External validation set display, the cross-center adaptability verification of our model is good, maintaining an AUC = 0.918 (95%CI: 0.876-0.959), which is significantly higher than the external verification of the pregnancy rate prediction model constructed by Loendersloot et al. (33) using multivariable logistic regression (AUC = 0.68), indicating that the model has strong predictive ability for ICSI-ET assisted pregnancy outcomes in infertile men and can effectively distinguish between patients who have achieved clinical pregnancy and those who have not. The calibration curve is used to evaluate the consistency between the predicted and actual risks of the model. Through the calibration chart, we observed a high degree of fit between the predicted and actual risks, further confirming the reliability of the model. The DCA curve further evaluated the practical application value of the model in clinical decision-making. In our study, the DCA curve of the external validation group showed superior net benefits of the model within the threshold probability range of 0-83%, indicating that using a nomogram model to assist clinical decision-making for ICSI-ET assisted pregnancy in infertile men is beneficial within this range. In addition, the DCA curves of each dataset model in this study consistently remained showed that the DCA curve shows that the net benefit curve of this model always stays above the two limits of “complete intervention” and “complete nonintervention”, indicating that using this model to guide clinical decision-making within the decision-making scope is effective and can bring the maximum clinical net benefit, indicating that it has certain advantages in practical clinical decision-making. This provides strong support for medical personnel in practical clinical decision-making, helping them better identify and manage the risks involved in assisted reproductive technology for infertile men. At the level of clinical translation, the related risk factors can be displayed in a line segment manner to achieve visualization and be integrated into a scoring system to realize the individualized prediction of the outcome of assisted reproduction in male infertility patients. Medical staff can assign values according to relevant clinical indicators, the higher the score, the better the outcome of assisted reproduction, and the operation is simple in clinical application, which can assist clinical decision-making. Medical personnel can implement corresponding intervention measures based on this predictive model, such as inviting nutrition and psychology departments to collaborate in developing intervention plans before entering the ICSI-ET assisted reproductive cycle, such as smoking cessation support, sleep cognitive-behavioral therapy, and developing exercise plans. After a period of behavioral and lifestyle changes, ICSI assisted reproductive cycle can be performed. Although the model of this study has good predictive value, the results of this study can only be used for auxiliary screening, and the final outcome of assisted reproduction still needs to be confirmed by clinical laboratory assistance.

### 4.5 Limitation

However, this study still has certain limitations. Firstly, cross-sectional surveys indeed have difficulty in clarifying the direction of causality. In the future, longitudinal data can be used to construct a cross-lag model to compare the path strength of the impact on assisted reproductive outcomes. Secondly, although this study eliminated some female factors that may have an impact on assisted reproductive outcomes, some factors such as oocyte quality and psychological status have not yet been included; what is more, the psychological scale used in this study was completed on the day of egg retrieval and reflects “status” rather than “traits”, which may lead to classification bias. In addition, the subjects of this study are infertile patients receiving ICSI for assisted reproduction, with a clear diagnosis of infertility, which may reduce or not apply to the predictive efficacy for other non-infertile male assisted reproduction patients. In the future, prospective impact exploration can be conducted to explore the three-level intervention pathways of lifestyle or antioxidants, and to review this predictive model to demonstrate its effectiveness.

## 5 Brief summary

This study established and validated a predictive model for the early clinical pregnancy outcomes of infertile men undergoing ICSI, consisting of 9 risk factors. These factors include both laboratory indicators such as semen parameters and related lifestyle factors, demonstrating significant predictive value and clinical application efficiency. Through the constructed nomogram, one can directly obtain the specific probability values of whether infertile men undergoing ICSI will achieve clinical pregnancy. This aids medical staff in clinical decision-making, providing a basis for comprehensively evaluating the semen quality and lifestyle of the infertile population, adopting proactive intervention measures to improve clinical outcomes, and formulating individualized medical plans, thus holding certain clinical significance.

## Data Availability

The original contributions presented in the study are included in the article/supplementary material. Further inquiries can be directed to the corresponding authors.
